# Toll-Like Receptors Expression in Follicular Cells of Patients with Poor Ovarian Response

**Published:** 2014-07-08

**Authors:** Seyed Abdolvahab Taghavi, Mahnaz Ashrafi, Mehdi Mehdizadeh, Leili Karimian, Mohammad Taghi Joghataie, Reza Aflatoonian

**Affiliations:** 1 Department of Anatomy, School of Medicine, Iran University of Medical Sciences, Tehran, Iran; 2 Department of Obstetrics and Gynecology, School of Medicine, Iran University of Medical Sciences, Tehran, Iran; 3 Department of Endocrinology and Female Infertility at Reproductive Biomedicine Research Center, Royan Institute for Reproductive Biomedicine, ACECR, Tehran, Iran; 4 Cellular and Molecular Research Center, Iran University of Medical Sciences, Tehran, Iran; 5 Department of Embryology at Reproductive Biomedicine Research Center, Royan Institute for Reproductive Biomedicine, ACECR, Tehran, Iran

**Keywords:** TLR, Ovary, Innate immunity, Infertility, MIF

## Abstract

**Background:**

Poor ovarian response (POR) to gonadotropin stimulation has led to a significant decline in success rate of fertility treatment. The immune system may play an important role in pathophysiology of POR by dysfunctions of cytokines and the growth factor network, and the presence of ovarian auto-antibodies. The aim of this study is to investigate the expression of toll-like receptors *(TLR) 1, 2, 4, 5, 6 and cyclooxygenase (COX) 2* genes in follicular cells and concentration of interleukin (IL)-6, IL-8 and macrophage migration inhibitory factor (MIF), as major parts of innate immunity, in follicular fluid (FF) obtained from POR women in comparison with normal women.

**Materials and Methods:**

In this case-control study, 20 infertile POR patients and 20 normal women took part in this study and underwent controlled ovarian stimulation. The FF was obtained from the largest follicle (>18 mm). The FF was centrifuged and cellular pellet was then used for evaluation of expression of *TLRs* and *COX2* genes by real-time PCR. FF was used for quantitative analysis for IL-6, IL-8 and MIF by enzyme-linked immunosorbent assay (ELISA).

**Results:**

*TLR1, 2, 4, 5, 6* and *COX2* gene expression were significantly higher in POR (p<0.05). Concentration of IL-6, IL-8 and MIF proteins was significantly increased in POR compared with normal women (p<0.05).

**Conclusion:**

These findings support the hypothesis that the immune system may be involved in pathophysiology of POR through TLRs.

## Introduction

A considerable issue in assisted reproductive technology (ART) is poor ovarian response (POR) to gonadotropin stimulation. This condition affects approximately 9 to 24% of the *in vitro* fertilization (IVF) cycles (1). POR leads to cycle cancellation, significant decline in number of oocyte and embryo, and reduction in the success rate of fertility treatment (2). POR is challenging to define, however, investigators have defined POR in patients that have a peak of E2 level <300 pg/ml, fewer mature oocytes and lower pregnancy rates after standard stimulation by human menopausal gonadotropin (3). POR is associated with advanced age, previous ovarian surgery, pelvic adhesion and high body mass index (BMI). However, young women are also affected by unanticipated poor response (1). Numerous hypotheses, although controversial, have been suggested for POR including poor follicular blood flow (4), dysfunctions of cytokines and the growth factor network (5), and the presence of ovarian auto-antibodies (6).

The immune system may play an important role in pathophysiology of POR. A major part of immune system is innate immunity that generates more rapid and primary responses to pathogens than the adaptive immune system (7). Key mediators of the innate immune system are toll-like receptors (TLRs) (8). To date, ten functional TLRs have been identified in the human genome. They are classified based on their cellular location; cell membrane TLRs (TLR1, 2, 4, 5, 6, 10) and endosomal TLRs (3, 7, 8, 9). In the current study we investigate the expression of cell membrane TLRs except TLR10, because its specific ligand is not known. These TLRs identify various pathogen associated molecular patterns (PAMPs) and damage associated molecular pattern (DAMPs), which activate a variety of host responses (9). TLR2 can form heterodimers with TLRs 1 and 6 and recognizes peptidoglycan, lipoteichoic acid and lipoarabinomannan resultant from pathogens including mycobacteria and zymosan from fungi and yeast (10), and endogenous ligands such as heat shock protein (HSP) -60, 70 and 96 (11), and reactive oxygen species (ROS) (12). TLR4 recognizes the lipopolysaccharide (LPS) as a main constituent of the outer membrane of Gram-negative bacteria, (13), Hyaluronan (14), HSPs (15) and Fibronectin(16). Bacterial flagellin is recognized via TLR5 (17).

Previous studies have discussed the functional TLRs in the reproductive system. TLRs 1-10 are expressed in the female reproductive tract (18, 19) and their expression level varies in different phases of the menstrual cycle (20).

TLRs are activated by specific ligands and can create intra-cellular signals via MYD88-dependent and independent pathways (9). These signals lead to induce gene expression of some inflammation related enzymes such as cyclo-ocygenase (COX) 2 which is a key enzyme in the conversion of arachidonic acid to prostaglandins (21). COX2 transcript has been identified in granulose cells and protein is present in pre-ovulatory follicular fluid (22, 23). Previous study has shown that COX2 negative follicles were anovulatory follicles (24). It is therefore concluded that COX2 has a central role in ovulation (24).

Moreover activation of TLRs leads to the stimulation of chemokine and cytokine expression including interleukin (IL) - 6, IL-8 and macrophage migration inhibitory factor (MIF) that activate a variety of host responses (9). These cytokines modulates systemic and local inflammatory and immune responses (25). Aboussahoud et al. demonstrated that TLRs expressed in endometrial epithelial cell lines and their stimulation led to IL-6 and IL-8 production (26).

Follicular cells play an important role in folliculogenesis, steroidogenesis and oocyte maturation (27). Previous studies have shown that ovarian follicular cells have innate immune capability (28) and express TLRs; Shimida et al. reported that mouse granulosa cells express TLR2, 4, 8 and 9 and are involved in cytokine and chemokine production (29). Zhou et al. (30) also suggested that the surface epithelium of human ovaries has high expression of TLR2-5.

Therefore, regarding the involvement of the immune system in POR pathogenesis (6, 31), the aim of the present study was to investigate the expression changes of *TLRs* and *COX2* genes in follicular cells as well as the concentration of MIF, IL-6 and IL-8 proteins in follicular fluid obtained from POR patients.

## Materials and Methods

### Patient characteristics

This case-control study was approved by Iran University of Medical Sciences and Royan Institute Ethics Committees. Forty participants (20 infertile poor ovarian responder patients and 20 normal women with male factor infertility as control) attending the Reproductive Medicine Unit in Royan Infertility clinic, Tehran, Iran, for intracytoplasmic sperm injection (ICSI) treatment were invited to participate in the study during 2012. An information sheet was offered to all women, and informed written consent was obtained.

Inclusion criteria were infertile women aged 20-35 undergoing ICSI treatment, receiving the same standard long protocol. The exclusion criteria were endometriosis, polycystic ovarian syndrome, endocrine disorder like hyperprolactinemia, history of ovarian surgery and female reproductive tract infection.

POR were defined as any patient who had abnormal ovarian reserve test [antral follicle count (AFC) <5-6 follicle or anti-Mullerian hormone (AMH) <0.5-1.1ng/ml] and a previous poor response (<3 oocyte retrieved) in control ovarian hyperstimulation (COH).

Anthropometric measurements were taken, including BMI [BMI, calculated as weight/(height)2 (kg/m^2^)]. Luteinizing hormone (LH), follicle stimulating hormone (FSH), anti mullerian hormone (AMH) and testosterone levels were determined. All laboratory parameters were determined in the early follicular phase of the menstrual cycle.

### Protocol for controlled ovarian stimulation

Patients underwent a standard long protocol using GnRH-a (Superfact, Aventis, Frankfurt, Germany) at a daily dose of 0.5 mg subcutaneous start on the day 17-19 of the natural menstrual cycle as a pre-treatment. Once pituitary desensitization was confirmed (endometrial thickness <5 mm and serum estradiol level <50 pg/ml), the GnRH-a dose was reduced by one-half and ovarian stimulation was initiated.

In all patients, ovarian stimulation started with a dose of 150-225 IU r-FSH (Gonal-F, Merck Serono, Switzerland) depending on the age of the patient. It was continued until the day of ovulatory human chorionic gonadotropin (hCG) administration according to the ovarian response. When at least two follicles were greater than 18 mm, 10,000 IU urinary hCG (Choriomon, IBSA, Lugano, Switzerland) was administered intramuscularly for ovulation induction and oocyte retrieval was performed 34-36 hours later (32).

### Sample collection

Follicular fluid aspiration was carried out with transvaginal ultrasound guidance using an aspiration needle from the largest follicle (>18mm) without flushing medium and blood contamination. The follicular fluid was transferred to a sterile Petri dish, and after the oocytes were removed, the fluid was located into a 15-mL conical tube and centrifuged at 300 g for 5 minutes. Supernatant was then removed.

### RNA isolation, cDNA production and RT-PCR

**Fig 1:**
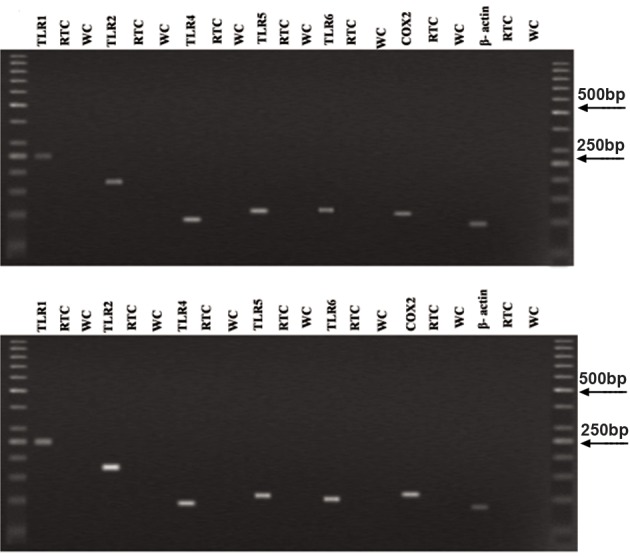
Result of RT-PCR for TLR1, 2, 4, 5, 6, COX2 and β-actin mRNA expression in control (A) and POR (B) groups. RTC; RT control, WC; Water control and COX; Cyclooxygenase.

One milliliter of TRI reagent (Sigma, Pool, UK) was added on the cellular pellet and homogenized for total RNA extraction following a standard protocol according to manufacturer’s instructions. Obtained total RNA in both groups was treated three times with DNase I (fermentase, sanktleon-rot, Germany) to remove genomic DNA contamination from the samples. First strand cDNA synthesis was performed using oligodT primers and reverse transcription by Super-Script II (Fermentas). Negative controls were prepared without addition of the enzyme (non-reverse transcribed controls, RT controls). The RT-PCR was performed using cDNA of each patient, Platinum Blue PCR Super Mix (Invitrogen, Pairsley, UK) and the forward and reverse primers for *TLR1, 2, 4, 5, 6,* and *COX2* (Metabion, martinsried, Germany). The forward and reverse primer sequences used are given in table 1. High cycle PCR allow us to identify the genes with low level of expression. The amplification was persistent for 40 cycles under the following setting: 95˚ for 30 seconds, 60-63˚ (Table 1) for 30 seconds, and 72˚ for 30 seconds. All experiments included RT controls as negative controls (no cDNA) and water control. PCR products were separated on 1.2% agarose gel. The amplified PCR products were sequenced to confirm the identity of amplified products. β-actin was used as a housekeeping control and its expression was checked between the two groups. Its expression was not different in POR and control groups (Fig 1).

**Table 1:** list of primers were used for regular PCR and real time –PCR


Variables	Forward primer(5-3)	Reverse primer(3-5)	Annealing temperature(C)	Product size(bp)

**TLR1**	GGGTCAGCTGGACTTCAGA	AAAATCCAAATGCAGGAACG	63	250
**TLR2**	TCGGAGTTCTCCCAGTTCTCT	TCCAGTGCTTCAACCCACAA	60	175
**TLR4**	TGATGTCTGCCTCGCGCCTG	AACCACCTCCACGCAGGGCT	60	98
**TLR5**	CACCAAACCAGGGATGCTAT	CCTGTGTATTGATGGGCAAA	60	111
**TLR6**	GCCACCATGCTGGTGTTGGCT	CGCCGAGTCTGGGTCCACTG	60	101
**COX2**	CAGCCATACAGCAAATCCT	TCTCCATAGAATCCTGTCCG	60	113
**β-actin**	CAAGATCATTGCTCCTCCTG	ATCCACATCTGCTGGAAGG	60	90


Sequence of TLRs, COX2 and β-actin primers used in the current investigation in RT-PCR. TLR; Toll-like receptor and COX; Cyclooxygenase.

### Quantitative real time PCR (QPCR)

QPCR was performed with the cDNA prepared from follicular cell pellet. QPCR reactions were carried out in triplicates using an ABI Prism 7300 Sequence Detector (Applied Biosystems, foster, USA) in a total volume of 20 μl containing 250 ng cDNA, 5 pmol gene specific primers and SYBR Green reagent (Applied Biosystems) with ROX dye as passive control for signal intensity. The thermal cycle profile followed 50 cycles at 95˚for 30 seconds, 60-63˚ (Table 1) for 30 seconds, and 72˚ for 30 seconds. Samples were run in triplicates. Melting curve analysis permitted determination of the specificity of the PCR fragments. All melting curves yielded one peak per PCR product. Standard curves were obtained using the logarithmic dilution series of total RNA.

The QPCR data were analyzed using the comparative CT method (33). In brief, the difference in cycle time (ΔCT) was determined as the difference between the number of cycles required for amplification of the test gene and the reference housekeeping gene, human *β-actin*. We then obtained ΔΔCT by finding the difference between groups. The fold change (FC) was calculated as -2^-ΔΔCT^.

### Immunoassay

Obtained FF from each patient was centrifuged at 300 g for 5 minutes, then supernatant was used to determine the concentration of IL-6, IL-8 and MIF by commercially Enzyme-Linked Immunosorbent Assay (ELISA) kits for IL-6 (eBioscience, Vienna, Austria), IL-8 (eBioscience, Vienna, Austria) and MIF (glory science, TX, USA).

Briefly, this technique uses a microwell plate coated with monoclonal antibody to human IL-6 (eBioscience), human IL-8/NAP-1 (eBioscience), human MIF (glory science), biotin-conjugate anti human IL-6 monoclonal antibody (eBioscience), biotin-conjugate anti human IL-8/NAP-1 monoclonal antibody (eBioscience), biotin-conjugate anti human MIF monoclonal antibody (glory science), streptavidine- HRP and tetramethyl-benzidine as a substrate.

Color change is measured spectrophotometrically at a wavelength of 450 nm. The concentration of these cytokines in the samples is then determined by comparing the O.D. of the samples to the standard curve.

Cytokine concentration were considered zero, if the detected cytokine concentration was equal to or less than the lower limit of kits (IL-6: 0.92 pg/ml, IL-8: 2.0 pg/ml and MIF: 0.003 pg/ml).

### Statistics

The results were expressed as mean ± SEM. Statistical analysis was performed by using t test in SPSS 18 software. P<0.05 was considered significant.

## Results

Clinical characteristics of the patients are presented in table 2.There is a significant difference in AMH, number of mature oocyte and total rFSH dose (IU) between POR and control groups.

**Table 2:** Clinical characteristics


P value	Control Group (N=20)	POR Group (N=20)	Variable

**Age (Y)**	30.47 ± 4.62	30.75 ± 3.89	0.727
**BMI (kg/m^2^),**	25.87 ± 2.92	25.20 ± 4.19	0.55
**Duration of infertility (Y)**	9.76 ± 6.04	7.70 ± 5.41	0.25
**Menstrual type, n (%)**			
**Regular**	20 (100)	20 (100)	
**Irregular**	0	0	
**LH (mU/ml)**	4.23 ± 2.24	5.02 ± 3.39	0.38
**FSH (mU/ml)**	10.99 ± 3.24	8.55 ± 5.37	0.08
**LH/FSH ratio**	0.56 ± 0.92	0.64 ± 0.33	0.71
**Testosterone (ng/ml)**	1.21 ± 0.31	1.31 ± 0.54	0.33
**AMH (ng/ml)**	0.175 ± 0.03	0.63 ± 0.18	0.02
**No. of mature oocyte**	1.5 ± 0.44	10.1 ± 1.59	0.00
**Total rFSH dose (IU)**	3321 ± 243.65	2390.3 ± 190.39	0.005


Presented as mean ± SD and compared by t test. BMI; Body mass index, AMH; Anti-mullerian hormone, LH; Luteinizing hormone, FHS; Follicle stimulating hormone and *; P<0.05. Clinical characteristics of the patients. There is significant difference in AMH, No. of mature oocyte and total rFSH dose (IU) between POR and control groups. Data were analyzed by t test.

Figure 1 shows the results of RT-PCR for mRNA expression of *TLRs* and *COX2* genes in human follicular cells in both groups. All amplified products had the expected size for that particular gene. There was no product amplified innegative control indicative of the lack of genomic DNA contamination.

**Fig 2:**
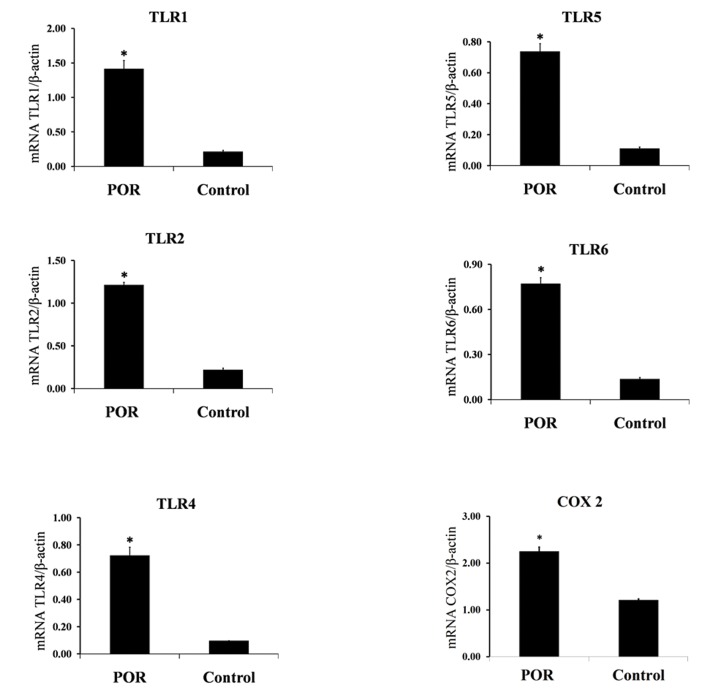
QPCR was used to quantify the expression of TLR1, 2, 4, 5, 6 and COX2 mRNA in POR and control groups. Data are presented as mean ± SEM of normalized expression values against internal controls (β-actin mRNA) in POR and control. TLR1, 2, 4, 5, 6 and COX2 showed a significantly higher expression in POR patients compared with the normal women. Data were analyzed by t test. *; P<0.05, POR; Poor ovarian response, MIF; Migration inhibitory factor, TLR; Toll-like receptor and COX; Cyclooxygenase.

The quantitative expression profiles of *TLR* genes in follicular cells in both groups are shown in figure 2. *TLR1, 2, 4, 5, 6* and *COX2* showed a significantly higher expression in POR patients compared to the control (p<0.05).

**Fig 3:**
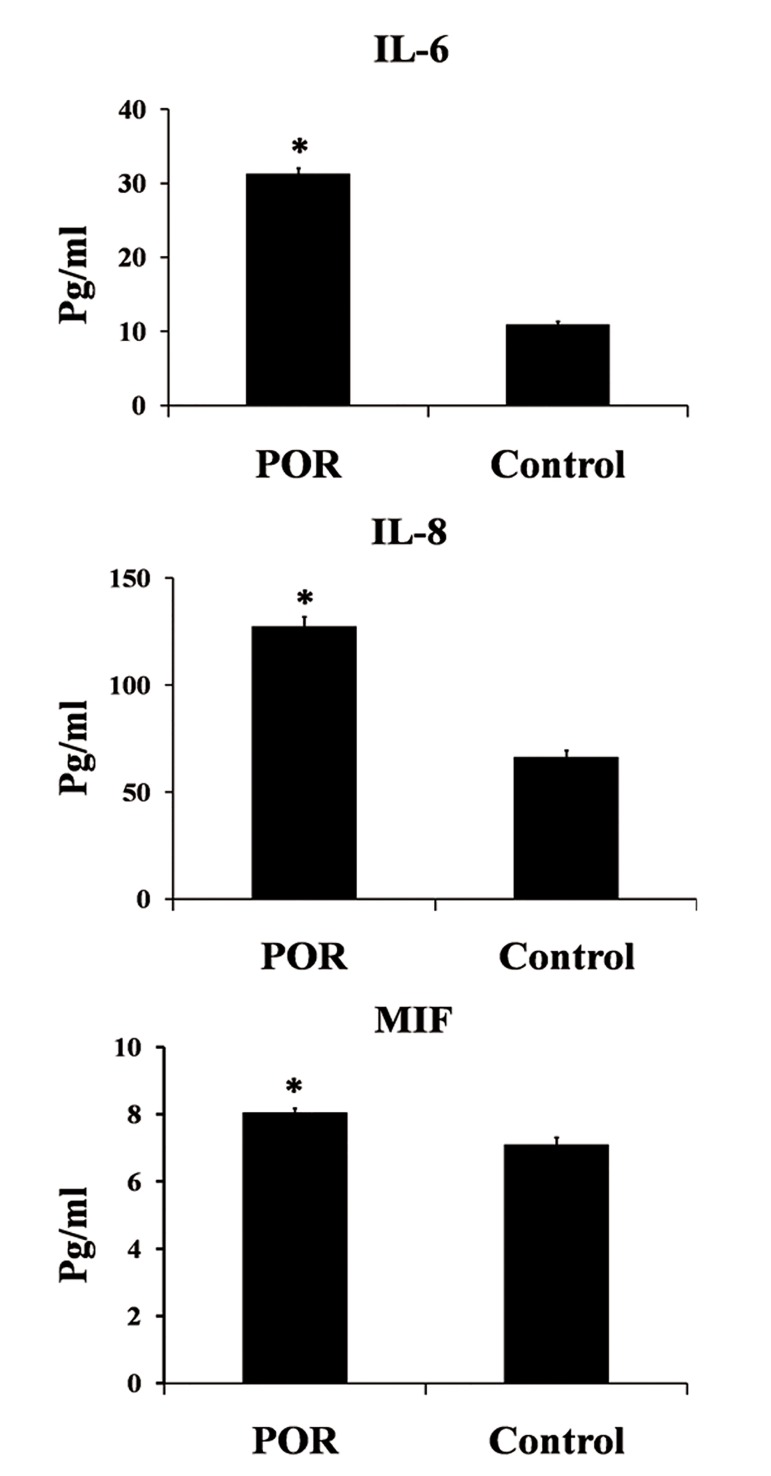
IL-6, IL-8 and MIF protein concentration obtained by ELISA in FF of POR and control groups. IL-6, IL-8 and MIF showed a significantly higher expression in POR compared with the control. Data were analyzed by t test. *; P<0.05, POR; Poor ovarian response, MIF; Migration inhibitory factor and IL; Interleukin.

The quantitative analysis of IL-6, IL-8 and MIF concentrations in FF by ELISA are shown in figure 3. IL-6, IL-8 and MIF were significantly increased in POR compared with control (p<0.05).

## Discussion

We found higher expression of *TLR1, 2, 4, 5* and 6 in POR compare to normal participants. Our data suggest that elevated expression of TLRs is correlated with the POR to standard ovarian stimulation protocol.

Numerous factors affect ovarian response to gonadotropin stimulation. It is suggested that the main reason of POR is diminished ovarian reserve (34). However, some young women with normal ovarian reserve present a poor response to ovarian stimulation (1). A hypothesis has been proposed for these patients: dysfunction of cytokines and the growth factor network (5) as a product of TLR signaling (35). Therefore, in the present study we investigated the expression of cell membrane TLRs in follicular cells of POR patients.

Our findings show that *TLR 1, 2, 4, 5, 6* were expressed in follicular cells of the control group, consistent with a recent study which showed COV434 human granulosa cell line expresses *TLR4-10* (36).

However, in relation to the TLR overexpression in POR patients, several hypotheses are discussed; i. TLR overexpression is a consequence of the presence of their endogenous or exogenous ligands in FF. Keay et al. (37) have reported a significantly higher prevalence of serum IgG antibodies to *C. trachomatis* in poor responders. Darville et al. (38) showed that *C. trachomatis* engages TLR2. ii. This expression pattern may be due to a susceptible genetic background in these POR patients where excessive TLR activation could be involved in the pathogenesis of POR through several mechanisms.

TLR activation leads to apoptosis through the Fas associated death domain (FADD) (39). Therefore increased follicular cell apoptosis leads to folliculogenesis impairment. Also, TLR activation results in excessive expression of COX-2 as a key mediator in ovulation (40). For instance Fukata et al. (41) demonstrated that TLR4 activation leads to COX-2 induction. Consistent with this study, our findings have shown that COX-2 expression is significantly higher in POR subsequent to TLR4 overexpression. COX-2 is a key enzyme in the conversion of arachidonic acid to prostaglandins (40). COX2 is upregulated in response to cytokines, growth factors, and estradiol stimuli. Likewise, it has been suggested that this enzyme is mostly associated with the inflammatory response (21).

TLRs have important role in cytokines production and autoimmunity (42). In agreement with this, our findings suggest that the overexpression of TLRs in follicular cells and excessive production of IL-6, IL-8 and MIF in follicular fluid simultaneously occur in the POR group. These cytokines affect the ovarian function and oocyte development through several mechanisms.

IL-6 diminishes aromatase activity within follicles which result in decreased intrafollicular E2 level, fertility and fertilizing capacity (43). IL-8 is a chemotactic activating cytokine for leukocytes and macrophages (44) with which their activation leads to increased ROS production (45). ROS bind to TLR 2 and 6 and consequently activate them (12).

Our study showed significant higher MIF protein production, consistent with significant *TLR4* over expression. This finding is in agreement with a recent study that showed TLR4 ligation leads to increased MIF level in epithelial ovarian cancer cells (46). MIF binding to CD74 stimulates pro-inflammatory cytokines, including IL-8, which leads to altered inflammatory and immune responses, cell proliferation and angiogenesis (47, 48).

Moreover, TLR signaling results in elevated levels of IL-10 in FF (49). It prevents p27 down-regulation in developing granulosa cells. Subsequently, G0 arrest of granulosa cell cycle leads to folliculogenesis impairment (50). Also, IL-10 is an anti-inflammatory cytokine and controls inflammation response via inhibiting TLR signaling pathways (51).

As previously stated, overstimulation of TLRs contribute to autoimmune response and tissue injury (42). Besides, a high correlation between POR and the presence of ovarian auto-antibody was seen (52, 53). These autoimmune responses primarily targets theca and granulosa cells (54), yielding dramatically reduced FSH receptor (FSHR) in POR. In a previous study, it has been shown that relative quantity of FSHR is positively correlated with two markers of ovarian response including number of mature oocytes and the peak level of serum E2 (3). Moreover, it has been stated that serum anti-FSH antibodies are increased in POR (55). FSH-FSHR interaction elicits intracellular signaling pathways responsible for proliferation and differentiation of granulosa cells. Subsequently, the FSH-stimulated granulosa cells produce E2 and sufficient E2 within the developing follicles further sustains oocyte development and maturation (56). Therefore, activation of TLRs may disturb the FSH-FSHR interaction which leads to poor proliferation and differentiation of granulosa cells and also reduced production of E2.

Despite the study being well-designed, the present study possesses the following limitations:

i. The number of included subjects was small and ii. It is a fact that POR group received increased volume of gonadotropins; therefore this increased dosage may have affected the immunological mechanisms.

## Conclusion

The association between increased TLR expression in follicular cells in POR suggest that TLRs may play important roles in the pathophysiology of POR. Further studies should be performed in future to confirm these findings and to determine the extent TLRs or other components of the TLR signaling pathway contribute to POR.
